# Patient-Reported Social and Behavioral Determinants of Health and Estimated Risk of Hospitalization in High-Risk Veterans Affairs Patients

**DOI:** 10.1001/jamanetworkopen.2020.21457

**Published:** 2020-10-20

**Authors:** Donna M. Zulman, Matthew L. Maciejewski, Janet M. Grubber, Hollis J. Weidenbacher, Dan V. Blalock, Leah L. Zullig, Liberty Greene, Heather E. Whitson, Susan N. Hastings, Valerie A. Smith

**Affiliations:** 1 Center for Innovation to Implementation, VA Palo Alto Health Care System, Menlo Park, California; 2 Division of Primary Care and Population Health, Stanford University School of Medicine, Stanford, California; 3 Durham Center of Innovation to Accelerate Discovery and Practice Transformation, Durham Veterans Affairs Health Care System, Durham, North Carolina; 4 Department of Population Health Sciences, Duke University, Durham, North Carolina; 5 Division of General Internal Medicine, Department of Medicine, Duke University, Durham, North Carolina; 6 Department of Medicine, Duke University School of Medicine, Durham, North Carolina; 7 Geriatric Research, Education, and Clinical Center, Durham Veterans Affairs Health Care System, Durham, North Carolina; 8 Center for the Study of Human Aging and Development, Duke University, Durham, North Carolina

## Abstract

**Importance:**

Despite recognition of the association between individual social and behavioral determinants of health (SDH) and patient outcomes, little is known regarding the value of SDH in explaining variation in outcomes for high-risk patients.

**Objective:**

To describe SDH factors among veterans who are at high risk for hospitalization, and to determine whether adding patient-reported SDH measures to electronic health record (EHR) measures improves estimation of 90-day and 180-day all-cause hospital admission.

**Design, Setting, and Participants:**

A survey was mailed between April 16 and June 29, 2018, to a nationally representative sample of 10 000 Veterans Affairs (VA) patients whose 1-year risk of hospitalization or death was in the 75th percentile or higher based on a VA EHR-derived risk score. The survey included multiple SDH measures, such as resilience, social support, health literacy, smoking status, transportation barriers, and recent life stressors.

**Main Outcomes and Measures:**

The EHR-based characteristics of survey respondents and nonrespondents were compared using standardized differences. Estimation of 90-day and 180-day hospital admission risk was assessed for 3 logistic regression models: (1) a base model of all prespecified EHR-based covariates, (2) a restricted model of EHR-based covariates chosen via forward selection based on minimizing Akaike information criterion (AIC), and (3) a model of EHR- and survey-based covariates chosen via forward selection based on AIC minimization.

**Results:**

In total, 4685 individuals (response rate 46.9%) responded to the survey. Respondents were comparable to nonrespondents in most characteristics, but survey respondents were older (eg, >80 years old, 881 [18.8%] vs 800 [15.1%]), comprised a higher percentage of men (4391 [93.7%] vs 4794 [90.2%]), and were composed of more White non-Hispanic individuals (3366 [71.8%] vs 3259 [61.3%]). Based on AIC, the regression model with survey-based covariates and EHR-based covariates better estimated hospital admission at 90 days (AIC, 1947.7) and 180 days (AIC, 2951.9) than restricted models with only EHR-based covariates (AIC, 1980.2 at 90 days; AIC, 2981.9 at 180 days). This result was due to inclusion of self-reported measures such as marital or partner status, health-related locus of control, resilience, smoking status, health literacy, and medication insecurity.

**Conclusions and Relevance:**

Augmenting EHR data with patient-reported social information improved estimation of 90-day and 180-day hospitalization risk, highlighting specific SDH factors that might identify individuals who are at high risk for hospitalization.

## Key Points

**Question:**

Can the estimation of risk of future hospitalization be improved by adding patient-reported social determinants of health to a model that is based on electronic health record data?

**Findings:**

In this survey study of 4685 respondent Veterans Affairs patients with 1-year risk in the 75th percentile or higher for hospitalization or death, a logistic regression model that included patient-reported social determinants of health outperformed a logistic model that was solely based on electronic health record variables.

**Meaning:**

Collecting social information from patients could improve health system algorithms that identify individuals at risk for poor outcomes.

## Teaser

This survey study describes social and behavioral determinants of health self-reported by Veterans Affairs patients who are at high risk for hospitalization and assesses whether adding these measures to a model based on electronic health record data improves the accuracy of estimates of 90-day and 180-day all-cause hospital admission risk.

## Introduction

There is increasing recognition that social and behavioral determinants of health (SDH)—the personal circumstances and environmental factors that shape individuals’ conditions of daily life—explain meaningful variation in clinical and economic outcomes.^1,2,3^ From a societal perspective, addressing SDH factors could increase the effectiveness of prevention and treatment efforts, health care use, and health expenditures.^4,5^ Clinician recognition of patients’ SDH vulnerabilities creates opportunities to offer social service referrals and targeted interventions for high-risk patients.^6,7,8^

In 2014, the Health and Medicine Division of the Institute of Medicine proposed that a battery of SDH measures be universally incorporated into electronic health records (EHRs) in an “interoperable fashion.”^9,10^ The Office of the Assistant Secretary for Planning and Evaluation of the Department of Health and Human Services reaffirmed this recommendation, based on an analysis that found that Medicare beneficiaries with social risk factors had worse outcomes than beneficiaries without them.^11^ Concurrently, a National Academies of Sciences, Engineering, and Medicine panel recommended identification and incorporation of a core set of SDH factors into federal payment reforms.^12^ To be clinically useful and merit integration into EHRs and payment reform, those measures must be brief, validated, and predictive of outcomes important to patients and health systems.

Because SDH measures are not routinely captured in most EHRs in general, little is known about which specific factors are most predictive of acute care use, such as hospitalization. Studies have found associations between hospitalizations and factors such as neighborhood socioeconomic status, race/ethnicity, food insecurity, and social isolation,^1,3,13,14,15^ but little is known about other social and behavioral characteristics, such as resilience and health-related locus of control.

The Veterans Affairs (VA) health care system has a long-standing mission to address SDH factors in its patient population,^16^ and VA users typically experience greater medical, social, and behavioral complexity than nonveterans or veterans not engaged in Veterans Administration care.^17,18^ With 2 decades of experience with a national EHR for millions of patients in the nation’s largest integrated health system, the VA is well positioned to inform selection and integration of SDH measures into EHRs. We leveraged this long-standing EHR by fielding a mailed survey with an array of SDH measures to a nationally representative sample of 10 000 VA patients who were at high risk for hospitalization. We linked the survey data to EHR data to describe variation in SDH factors and to assess whether adding patient-reported SDH measures to models with EHR-based covariates improves estimation of 90-day and 180-day all-cause hospital admission risk.

## Methods

### Conceptual Model and Measure Selection

Survey measures were informed by the Cycle of Complexity model,^19^ which posits that patient complexity is a multifactorial construct composed of workload, acute shocks and medical events, and capacity or resilience, all of which influence access or use. Study team members (D.M.Z., M.L.M., J.M.G., H.J.W., D.V.B., L.L.Z., H.E.W., and S.N.H.) used an iterative consensus process to generate a draft list of survey measures, prioritizing measures that were not available in administrative data, had a known association with hospitalization, explained the maximum amount of variation within the construct, shared minimal overlap with other scales, and could help primary care teams identify patients who would benefit from clinical intervention. This study followed the American Association for Public Opinion Research (AAPOR) reporting guideline for survey studies. This evaluation was reviewed and designated as nonresearch quality improvement by the supporting Veterans Health Administration program office and the Durham VA Medical Center institutional review board and therefore did not require approval or informed consent. Per VA protocol, individuals who were invited to participate were sent a letter describing the survey's purpose and inviting them to opt out by mail or telephone if they did not wish to participate or to be contacted again. Returning the survey constituted consent.

The final survey included measures of educational attainment, employment, economic circumstances, smoking status,^20^ global health status (SF-1^21,22^), functional status (activities of daily living [ADL], instrumental ADL [IADL]^23^), and sleep quality.^24^ Measures of health literacy^25^ and patient activation from the Consumer Health Activation Index^26^ were included because these characteristics may help determine the level of assistance that patients need with health care navigation. Social circumstances could be critical in helping a patient avoid acute care use; thus, the survey also queried patients about their marital or partner status (“Are you currently married or living with a partner?”), social support (Medical Outcomes Study Social Support Survey 8^27^), and loneliness.^28^ Factors that reflect socioeconomic status included transportation availability and medication and food insecurity.^29^ Depression symptoms (2-item Patient Health Questionnaire^30^), resilience,^31^ Multidimensional Health Locus of Control,^32^ chaotic lifestyle (CHAOS^33^), recent life stressors (adapted from Duke Established Populations for Epidemiologic Studies of the Elderly [EPESE]^34^), and grit^35^ were assessed because these factors may be important risk factors for acute health deterioration and health care needs.

### Analytic Sample

The sampling frame included veterans with a 1-year risk of hospitalization or death on March 16, 2018, that was in the 75th or higher percentile based on the VA’s Care Assessment Need score,^36,37^ the lower end of which reflects approximately 10% to 11% probability of hospitalization or death in the upcoming year. The 1-year risk measure is more stable and should fluctuate over time more slowly than a 90-day risk measure. A sample of 10 000 high-risk veterans within US VA medical centers was selected, using PROC SURVEYSELECT (SAS, version 9.4; SAS Institute Inc), via stratified random sampling proportional to the percentage of veterans with Care Assessment Need score 75 or higher in each US VA medical center to obtain a nationally representative sample of veterans who had at least 1 VA outpatient visit from March 20, 2017, to March 18, 2018, were alive, and had a valid home address at the time of cohort generation.

The survey was submitted to the US Office of Management and Budget for review on June 13, 2016, and approved on October 25, 2017. A mail survey was fielded by Westat on April 16, 2018, to 10 000 veterans. Using the Dillman method,^38^ the survey was sent with a cover letter, a $2 bill incentive, a prepaid return envelope, and information for opting out from further mailings via a toll-free telephone number or return postcard. The cover letter described the purpose of the survey and included a statement that survey completion was voluntary and indicated consent to link survey responses to health record information. Veterans who did not opt out and did not respond within a 6-week period were mailed a second survey with a prepaid envelope and cover letter.

### VA Electronic Health Record Data

We merged survey respondents’ survey data with their EHR data from the VA’s Corporate Data Warehouse. Demographic characteristics included age, sex, race and ethnicity as reported in the EHR, VA copay status (a marker of socioeconomic status), and rural vs urban status of residence. Clinical characteristics included chronic conditions in the Gagne comorbidity score,^39^ and 7 conditions not included in the Gagne comorbidity score (anxiety, bipolar disorder, dyslipidemia, posttraumatic stress disorder, schizophrenia, traumatic brain injury, and traumatic spinal cord injury) that are common in veteran populations. These chronic conditions were identified based on at least 1 outpatient encounter with an *International Statistical Classification of Diseases and Related Health Problems, Tenth Revision* code for the condition in the year before the data pull. Other clinical characteristics extracted from the EHR included body mass index, frailty (JEN Frailty Index),^40^ and alcohol use based on the 3-item Alcohol Use Disorders Identification Test–Consumption (AUDIT-C).^41^

### Outcomes and Statistical Analysis

The primary outcomes of interest—all-cause VA hospitalization 90 and 180 days after survey completion (operationalized as 7 days before receipt by survey vendor)—were obtained from the VA EHR. To examine whether survey respondents differed from nonrespondents, we compared EHR-based characteristics (eg, age, sex, race/ethnicity, VA copay status, rural residence, comorbidities) between survey respondents and nonrespondents using standardized mean differences (SMDs), calculated using tableone in R.^42,43^ The use of SMDs are recommended by the American Statistical Association because, unlike *P* values, SMDs are insensitive to sample size,^44^ thereby protecting against overstatement of significance.^45^

Among respondents, we conducted 3 sets of logistic regressions for each of 2 dependent variables (90-day and 180-day all-cause hospitalizations) to identify specific patient-reported SDH measures that improved estimation of patient risk of hospital admission. Our goal was to evaluate whether including survey-based SDH measures improved estimates of hospitalization risk by comparing 3 models: (1) a base model of all prespecified EHR-based covariates (ie, all variables in Table 1, except individual Gagne conditions), (2) a restricted model of EHR-based covariates chosen via forward selection based on minimizing Akaike information criterion (AIC), and (3) a model of EHR- and survey-based covariates chosen via forward selection (including base model variables and all survey-based variables in Table 2, except number of stressful life events) based on AIC minimization.^46^ The AIC was used as the primary criteria for estimate accuracy, based on its ability to reflect model performance and because the rare outcome examined did not allow for withholding a validation sample.^46^ The AIC relates to *P* values, but with a penalty for model complexity, prioritizing a more parsimonious model that often leads to improved out-of-sample estimation, particularly when there are a small number of events (eg, hospitalizations) compared with the number of variables considered.^46,47^ Differences of AIC greater than 2 suggest considerable improvement; differences greater than 10 suggest substantial improvement.^47^ Although backward selection is generally preferred, forward selection was used because the sparseness of the binary outcome precluded entering all covariates into the model simultaneously.^48^ Because the goal of the analysis was to identify the model that yielded the most accurate estimates (not a model limited to statistically significant covariates), the final models could include covariates that were or were not statistically significant at a .05 threshold. We also report C statistics to facilitate interpretation of findings, although C statistics reflect in-sample classification, sorting those on which we already have data into one outcome or the other, rather than the ability of a model to predict an outcome on a patient not in the current sample. All continuous covariates were assessed for linearity using restricted cubic splines, as computed with the %PSPLINET SAS macro.^49^ Analyses other than calculation of standardized mean differences were conducted using SAS, version 9.4.

**Table 1.** Characteristics of Survey Respondents and Nonrespondents^a^

Characteristic	No. (%)		SMD^b^
	Respondent (n = 4685)	Nonrespondent (n = 5315)	
Demographic characteristics			
Age, y			
<60	642 (13.7)	1624 (30.6)	0.42
60-80	3162 (67.5)	2891 (54.4)	
>80	881 (18.8)	800 (15.1)	
Male	4391 (93.7)	4794 (90.2)	0.13
Race/ethnicity			
Non-Hispanic White	3366 (71.8)	3259 (61.3)	0.25
Non-Hispanic Black	741 (15.8)	1298 (24.4)	
Hispanic	203 (4.3)	289 (5.4)	
Other	103 (2.2)	149 (2.8)	
Missing	272 (5.8)	320 (6.0)	
VA copay status			
Nonexempt	584 (12.5)	614 (11.6)	0.02
Exempt	2988 (63.8)	3314 (62.4)	
Missing	1113 (23.8)	1387 (26.1)	
Rural residence	1732 (37.0)	1596 (30.1)	0.15
Missing	3 (0.1)	5 (0.1)	
Chronic health conditions			
Gagne comorbidity score, mean (SD)^c^	1.64 (1.93)	1.61 (1.96)	0.02
Individual Gagne conditions^c^			
AIDS/HIV	30 (0.6)	59 (1.1)	0.05
Alcohol abuse	472 (10.1)	851 (16.0)	0.18
Cardiac arrhythmias	1032 (22.0)	876 (16.5)	0.14
COPD	1340 (28.6)	1288 (24.2)	0.10
Coagulopathy	100 (2.1)	97 (1.8)	0.02
Congestive heart failure	657 (14.0)	619 (11.6)	0.07
Deficiency anemia	288 (6.1)	331 (6.2)	0.00
Dementia	203 (4.3)	393 (7.4)	0.13
Diabetes, complicated	1429 (30.5)	1383 (26.0)	0.10
Fluid and electrolyte disorders	321 (6.9)	351 (6.6)	0.01
Hypertension (uncomplicated or complicated)	3425 (73.1)	3366 (63.3)	0.21
Liver disease	316 (6.7)	475 (8.9)	0.08
Any malignancy^d^	948 (20.2)	837 (15.7)	0.12
Metastatic cancer	80 (1.7)	102 (1.9)	0.02
Paralysis (hemiplegia or paraplegia)	42 (0.9)	58 (1.1)	0.02
Peripheral vascular disorders	659 (14.1)	574 (10.8)	0.10
Psychoses	130 (2.8)	304 (5.7)	0.15
Pulmonary circulation disorders	96 (2.0)	110 (2.1)	0.00
Kidney failure	770 (16.4)	722 (13.6)	0.08
Weight loss	156 (3.3)	240 (4.5)	0.06
Non-Gagne health conditions			
Anxiety	605 (12.9)	979 (18.4)	0.15
Bipolar	169 (3.6)	301 (5.7)	0.10
Dyslipidemia	2609 (55.7)	2525 (47.5)	0.16
Posttraumatic stress disorder	854 (18.2)	1263 (23.8)	0.14
Schizophrenia	97 (2.1)	225 (4.2)	0.12
Traumatic brain injury	13 (0.3)	46 (0.9)	0.08
Traumatic spinal cord injury	33 (0.7)	47 (0.9)	0.02
Other clinical characteristics			
BMI			
<25	951 (20.3)	1274 (24.0)	0.11
25 to <30	1463 (31.2)	1523 (28.7)	
30 to <35	1195 (25.5)	1225 (23.0)	
≥35	899 (19.2)	973 (18.3)	
Missing	177 (3.8)	320 (6.0)	
Frailty (JEN Frailty Index), mean (SD)^e^	4.32 (1.77)	4.38 (1.91)	0.03
Alcohol Use (AUDIT-C score)			
None (0)	2801 (59.8)	3155 (59.4)	0.07
Low (1-2 [female], 1-3 [male])	1330 (28.4)	1454 (27.4)	
Moderate (3-7 [female], 4-7 [male])	392 (8.4)	444 (8.4)	
High (≥7)	106 (2.3)	175 (3.3)	
Missing	56 (1.2)	87 (1.6)	

Abbreviations: AUDIT-C, Alcohol Use Disorders Identification Test–Consumption; BMI, body mass index (calculated as weight in kilograms divided by height in meters squared); COPD, chronic obstructive pulmonary disease; SMD, standardized mean difference; VA, Veterans Affairs.

^a^ The variables in the table represent all the covariates included in the electronic health record–based logistic regression analyses; response levels are identical to those used in the regression analyses unless otherwise noted.

^b^ Standardized mean differences were calculated using the tableone package^43^ in R software, version 3.6.0.

^c^ Gagne score was included only in the primary logistic regression models and, owing to lack of linearity on the log scale, was included as a discontinuous continuous variable. In post hoc analyses, Gagne was replaced with individual Gagne conditions with nonzero weights.

^d^ Any type of malignant neoplasm, including lymphoma and leukemia, but excluding malignant neoplasm of the skin.

^e^ Index missing for 34 respondents and 48 nonrespondents.

**Table 2.** Patient-Reported Survey Responses (4685 Patients Responded)

Item	No. (%)	
	Responders	Missing values
Workload domain		
Global health status		145 (3.1)
Good, very good, or excellent	2168 (46.3)	
Poor or fair	2372 (50.6)	
Physical function: limitations in activities of daily living, mean (SD)	0.81 (1.51)	250 (5.3)
Physical function: instrumental activities of daily living (score ≥4)	817 (17.4)	277 (5.9)
Smoking status		231 (4.9)
Never	1113 (23.8)	
Past	2338 (49.9)	
Current, less than daily	210 (4.5)	
Current daily	793 (16.9)	
Transportation barriers present	868 (18.5)	187 (4.0)
Loneliness score ≥5	1941 (41.4)	237 (5.1)
Sleep quality (normalized t-score)		220 (4.7)
≤48	756 (16.1)	
>48 to 60	2587 (55.2)	
>60	1122 (23.9)	
Chaotic lifestyle score, mean (SD)	15.63 (4.13)	153 (3.3)
Medication insecure	570 (12.2)	146 (3.1)
Food insecure	962 (20.5)	279 (6.0)
Depression symptoms (PHQ-2 ≥3)	1271 (27.1)	254 (5.4)
Capacity/resilience domain		
Health literacy		
Lack confidence in completing medical forms	498 (10.6)	48 (1.0)
Had help completing survey	711 (15.2)	55 (1.2)
Employed (full time or part time)	574 (12.3)	84 (1.8)
Finances okay or good	2672 (57.0)	128 (2.7)
<High school graduate	436 (9.3)	40 (0.9)
Social support score		299 (6.4)
≤48	1200 (25.6)	
>48-84	1630 (34.8)	
>84	1556 (33.2)	
Patient activation score^a^		183 (3.9)
≤11	1413 (30.2)	
11 to <14	1833 (39.1)	
≥14	1256 (26.8)	
Low resilience (score ≤3)	1862 (39.7)	160 (3.4)
Grit		
Perseverance of effort (score ≥5)	136 (2.9)	224 (4.8)
Consistency of interest score		224 (4.8)
≤2	445 (9.5)	
>2-4	3183 (67.9)	
>4	833 (17.8)	
Married	2641 (56.4)	142 (3.0)
Acute shocks and medical events domain		
No. of stressful life events in past year^b^		191 (4.1)
0	834 (17.8)	
1	975 (20.8)	
≥2	2685 (57.3)	
Preference/expectations		
IHLC score		191 (4.1)
<18	879 (18.8)	
18-24	2689 (57.4)	
>24	926 (19.8)	
CHLC score ≥18	2632 (56.2)	219 (4.7)
PHLC score		185 (3.9)
≤18	1981 (42.3)	
>18 to 22	1623 (34.6)	
>22	896 (19.1)	

Abbreviations: CHLC, chance health locus of control; IHLC, internal health locus of control; PHLC, powerful others health-related locus of control; PHQ-2, 2-item Patient Health Questionnaire.

^a^ Based on 4 items: “I always know what steps to take when I have a health problem”; “I always know where to look for information before making decisions about my health”; “It is very easy for me to make changes to my daily life to improve my health”; and “It is very easy for me to follow my doctor’s instructions.”

^b^ This variable is included only in the post hoc analyses, not the primary outcome logistic regression. In post hoc analyses this variable was not retained in any of the by-imputation logistic regression models and, therefore, does not appear in post hoc analyses presented in eFigure 2, eTable 1, or eTable 2 in the Supplement.

Most survey and some EHR covariates had at least a small proportion (1%-6%) of missing data, but listwise deletion of respondents with missing values would have resulted in the loss of approximately 70% of respondents. Therefore, we multiply (n = 10) the imputed data using the MCMC option in PROC MI. The PROC HPLogistic procedure with forward selection based on minimizing AIC was used to run logistic regressions for each of the 10 imputed data sets and each of the 3 specifications for the 90-day and 180-day hospitalization outcomes. Logistic results for the second and third models (those using forward selection) were examined to determine the number of times that each covariate was chosen for inclusion in the final model. We identified covariates as important for improving estimates if they were chosen in at least 6 of 10 imputed data set models (eFigure 1 and eFigure 2 in the Supplement). After these covariates were identified, we entered them into a logistic regression model for each of the imputed data sets for both the 90-day and 180-day hospitalization outcomes using PROC Logistic (SAS, version 9.4). The regression results from the 10 imputations were combined using PROC MIANALYZE to appropriately estimate odds ratios and 95% CIs.

We also conducted post hoc analyses, repeating all of the above logistic regressions, but replacing the EHR-based Gagne comorbidity score with the individual condition indicators that underlie the score in all 3 models and adding in a survey-based measure of the number of life stressors experienced in the past year, adapted from the EPESE study,^34^ to the set of survey measures included in the EHR Plus Survey model.

## Results

There were 4685 individuals who completed the survey (response rate 46.9%, eFigure 3 in the Supplement). Respondents and 5315 nonrespondents were comparable across most (3 of 36) variables, but survey respondents were older (eg, >80 years old, 881 [18.8%] vs 800 [15.1%]), had a higher percentage who were male (4391 [93.7%] vs 4794 [90.2%]), were composed of more White non-Hispanic patients (3366 [71.8%] vs 3259 [61.3%]), and resided in a rural location (1732 [37.0%] vs 1596 [30.1%]). In addition, respondents and nonrespondents differed somewhat across a few individual clinical conditions (eg, hypertension, 3425 [73.1%] vs 3366 [63.3%]) (Table 1), hospitalization rate (492 [10.5%] vs 718 [13.5%]), and mortality rate (89 [1.9%] vs 282 [5.3%]) at 180 days. Respondent survey-based characteristics organized by Cycle of Complexity domains^19^ are presented in Table 2.

### Estimation of 90-Day Hospital Admission Risk

The unadjusted 90-day VA hospital admission rate among the survey respondents was 5.6%. Based on AIC scores (Table 3), the logistic regression that included both patient-reported and EHR-based covariates improved estimates of 90-day hospitalization (AIC, 1947.7) compared with regressions that only had a restricted (AIC, 1963.9) or full set (AIC, 1980.2) of EHR-based covariates, indicating a substantial (ie, AIC>10) improvement. The corresponding improvement in the C statistic from restricted-EHR to survey plus EHR models was 0.643 to 0.671. Estimation accuracy, relative to models using only EHR data, was improved by the additional inclusion of 8 SDH survey measures: marital or partner status, smoking status, resilience, powerful others health-related locus of control, medication security status, global health status, depression symptoms, and health literacy as indicated by patient-reported confidence in completing medical forms (Table 3).

**Table 3.** Logistic Regression Associations Between Covariates of Interest and 90-Day Hospital Admission, by Model Type (N = 4685)

Covariates	Odds ratio (95% CI)^a^		
	Model with EHR-based covariates		Model with EHR- and survey-based covariates, forward selection
	All	Forward selection	
EHR-based measure			
Demographic characteristic			
Age, y			
<60	1 [Reference]		
60-80	1.05 (0.70-1.59)		
>80	0.90 (0.54-1.51)		
Sex			
Male	1 [Reference]	1 [Reference]	1 [Reference]
Female	0.50 (0.24-1.04)	0.52 (0.25-1.07)	0.48 (0.23-1.00)
Race/ethnicity			
Non-Hispanic White	1 [Reference]		
Non-Hispanic Black	1.10 (0.78-1.55)		
Hispanic	1.63 (0.95-2.77)		
Other	1.16 (0.52-2.61)		
VA copay status			
Exempt	1 [Reference]	1 [Reference]	
Nonexempt	0.74 (0.49-1.12)	0.71 (0.47-1.08)	
Residence			
Nonrural	1 [Reference]		
Rural	0.88 (0.67-1.16)		
Chronic health condition			
Gagne comorbidity score^b^			
For 1-unit increase (when initial score in −2 to 0 range)	0.82 (0.48-1.39)	0.84 (0.50-1.43)	0.84 (0.49-1.43)
For 1-unit increase (when initial score in 1 to 13 range)	1.09 (0.63-1.89)	1.08 (0.63-1.88)	1.08 (0.62-1.87)
Non-Gagne health condition (presence vs absence [reference])			
Anxiety	0.95 (0.65-1.38)		
Bipolar disorder	1.80 (0.99-3.28)	1.75 (0.98-3.14)	1.71 (0.94-3.08)
Dyslipidemia	0.86 (0.66-1.11)		
Posttraumatic stress disorder	0.68 (0.47-0.97)	0.70 (0.49-0.99)	0.74 (0.52-1.06)
Schizophrenia	1.99 (1.02-3.86)	2.09 (1.08-4.03)	2.00 (1.03-3.90)
Traumatic brain injury	2.03 (0.41-10.01)		
Spinal cord trauma	2.04 (0.69-6.05)		
Other clinical characteristic			
BMI			
<25	1 [Reference]		
25 to <30	1.00 (0.68-1.46)		
30 to <35	0.96 (0.65-1.44)		
≥35	1.09 (0.72-1.64)		
Frailty (JEN Frailty Index, for 1-unit increase)	1.23 (1.14-1.33)	1.24 (1.15-1.33)	1.23 (1.14-1.32)
Alcohol use (AUDIT-C score)			
None (0)	1 [Reference]	1 [Reference]	
Low (1-2 [female], 1-3 [male])	0.94 (0.70-1.27)	0.95 (0.71-1.28)	
Moderate (3-7 [female], 4-7 [male])	1.19 (0.75-1.91)	1.22 (0.76-1.94)	
High (>7)	2.14 (1.11-4.11)	2.23 (1.18-4.21)	
Survey-based measure			
Workload domain			
Global health status			
Poor/fair			1 [Reference]
Good/very good/excellent			1.36 (1.02-1.81)
Smoking status			
Never			1 [Reference]
Past			1.11 (0.79-1.55)
Current, less than daily			0.89 (0.45-1.75)
Current daily			1.63 (1.10-2.40)
Medication			
Secure			1 [Reference]
Insecure			0.63 (0.40-1.00)
Depression symptoms, PHQ-2			
0-2			1 [Reference]
≥3			1.33 (0.97-1.82)
Capacity/resilience domain			
Lack confidence in completing medical forms			1.32 (0.91-1.91)
Low resilience, score			
>3			1 [Reference]
≤3			1.16 (1.00-1.34)
Married			
No			1 [Reference]
Yes			0.69 (0.52-0.90)
Preference/expectations			
Powerful others health-related locus of control, score			
≤18			1 [Reference]
>18 to 22			1.02 (0.77-1.36)
>22 to 30			0.71 (0.49-1.04)
Mean AIC (SD)	1980.2 (1.7)	1963.9 (1.6)	1947.7 (2.5)

Abbreviations: AIC, Akaike Information Criterion; AUDIT-C, Alcohol Use Disorders Identification Test–Consumption; BMI, body mass index (calculated as weight in kilograms divided by height in meters squared); EHR, electronic health record; PHQ-2, 2-item Patient Health Questionnaire; VA, Veterans Affairs.

^a^ Calculated using PROC MIANALYZE within SAS software, version 9.4, to combine estimates from 10 imputed data sets (SAS PROC MI) per model type. Although the primary analyses were not based on statistical significance, the 95% CIs are provided to inform uncertainty around the magnitude of the estimates from the covariates selected as improving estimates of the outcomes based on AIC.

^b^ Gagne score was included in the model as a piecewise continuous variable composed of 2 pieces, one for Gagne scores with initial value of −2 to 0 and the other for Gagne scores with initial values of 1 to 13. The reported Gagne odds ratios and 95% CIs are for a 1-unit increase in the Gagne score.

In post hoc analyses that incorporated a categorized measure of the number of recent life stressors and replaced the Gagne score with individual condition indicators (eTable 1 in the Supplement), the logistic regression that included both patient-reported and EHR-based covariates also improved estimates of 90-day hospitalization (AIC, 1940.2) compared with regressions that had only a restricted set (AIC, 1954.0) or a full set (AIC, 1994.1) of EHR-based covariates. The 8 measures that improved the estimates of 90-day hospitalization in the primary analyses were the same ones that improved the estimates of the 90-day post hoc model (Table 4).

**Table 4.** Summary of SDH Variables Included in Final EHR Plus Survey–Based, 90-Day and 180-Day Primary and Post Hoc Logistic Regression Models^a^

Item	Variables selected for model in ≥6 of 10 imputations	
	90-d Hospitalization	180-d Hospitalization
Global health status	X^b^	
Smoking status	X^b^	
Medication insecure	X^b^	
Depression symptoms	X^b^	
Health literacy (lack of confidence completing forms)	X^b^	
Low resilience	X^b^	X^b^
Marital or partner status	X^b^	X^b^
Health locus of control: powerful others	X^b^	X
Chaotic lifestyle		X^b^
Physical function: ADL limitations		X^b^

Abbreviations: ADL, activities of daily living; EHR, electronic health record; SDH, social and behavioral determinants of health.

^a^ Measures in the table are the SDH measures that improved estimates of hospitalization in the final 90-day or 180-day analyses; measures with an X are those that were selected in ≥6 of 10 imputations (specific numbers are included in eFigures 1 and 2 in the Supplement).

^b^ SDH measure also improved estimates in the final post hoc analysis. No SDH measures emerged as improving estimates of hospitalization in the final post hoc analyses that had not already been identified as improving hospitalization in the same time frame in the final primary analyses.

### Estimating Risk of 180-Day Hospital Admission

The unadjusted 180-day VA hospital admission rate was 10.0%. Based on AIC scores, augmenting EHR covariates with patient-reported survey measures improved estimates of 180-day outcomes (AIC, 2951.9) compared with regressions with the restricted set (AIC, 2961.3) and the full set (AIC, 2981.9) of EHR-based covariates (Table 5). For the restricted models, the corresponding C statistics ranged from 0.636 to 0.651. Three of the same factors that emerged in the primary 90-day model also improved estimates of 180-day hospitalization (marital or partner status, resilience, and powerful others health-related locus of control), along with 2 others (activities of daily living and chaotic lifestyle) (Table 4). In post hoc analyses (eTable 2 in the Supplement), patient-reported survey measures also improved estimates of 180-day outcomes (AIC, 2941.4) over the restricted EHR model (AIC, 2949.9) and the full EHR model (AIC, 2994.8). Four of the 5 survey measures (marital or partner status, resilience, activities of daily living, and chaotic lifestyle) that improved estimates of 180-day hospitalization in the primary analysis were the same measures that improved estimates of 180-day hospitalization in the post hoc analysis.

**Table 5.** Logistic Regression Associations Between Covariates of Interest and 180-Day Hospital Admission, by Model Type (N = 4685)

Covariates	Odds ratio (95% CI)^a^		
	Model with EHR-based covariates		Model with EHR- and survey-based covariates, forward-selected
	All	Forward-selected	
EHR-based measure			
Demographic characteristic			
Age, y			
<60	1 [Reference]		
60-80	0.88 (0.65-1.19)		
>80	0.76 (0.51-1.11)		
Sex			
Male	1 [Reference]		
Female	0.75 (0.47-1.19)		
Race/ethnicity			
Non-Hispanic White	1 [Reference]		
Non-Hispanic Black	0.97 (0.74-1.28)		
Hispanic	1.06 (0.66-1.69)		
Other	1.28 (0.71-2.31)		
VA copay status			
Exempt	1 [Reference]	1 [Reference]	1 [Reference]
Nonexempt	0.69 (0.50-0.97)	0.69 (0.50-0.96)	0.72 (0.52-1.01)
Residence			
Nonrural	1 [Reference]	1 [Reference]	1 [Reference]
Rural	0.85 (0.69-1.05)	0.85 (0.69-1.05)	0.86 (0.70-1.06)
Chronic health condition			
Gagne comorbidity score^b^			
For 1-unit increase (when initial score in −2 to 0 range)	0.95 (0.63-1.44)	0.96 (0.63-1.46)	0.97 (0.64-1.47)
For 1-unit increase (when initial score in 1 to 13 range)	1.11 (0.72-1.70)	1.11 (0.72-1.70)	1.14 (0.74-1.77)
Non-Gagne health conditions (presence vs absence [reference])			
Anxiety	0.94 (0.71-1.26)		
Bipolar disorder	1.46 (0.90-2.35)	1.44 (0.91-2.28)	1.44 (0.90-2.29)
Dyslipidemia	0.99 (0.81-1.20)		
Posttraumatic stress disorder	0.88 (0.68-1.14)		
Schizophrenia	1.70 (0.98-2.95)	1.75 (1.01-3.01)	1.71 (0.98-2.96)
Traumatic brain injury	1.08 (0.22-5.24)		
Spinal cord trauma	1.72 (0.69-4.31)		
Other clinical characteristic			
BMI			
<25	1 [Reference]		
25 to <30	0.94 (0.71-1.26)		
30 to <35	0.93 (0.69-1.26)		
≥35	1.03 (0.76-1.41)		
Frailty (JEN Frailty Index, for 1-unit increase)	1.21 (1.14-1.28)	1.21 (1.14-1.28)	1.21 (1.14-1.28)
Alcohol use (AUDIT-C score)			
None (0)	1 [Reference]	1 [Reference]	1 [Reference]
Low (1-2 [female], 1-3 [male])	0.89 (0.71-1.12)	0.90 (0.72-1.13)	0.90 (0.71-1.14)
Moderate (3-7 [female], 4-7 [male])	1.30 (0.92-1.84)	1.33 (0.94-1.87)	1.32 (0.94-1.87)
High (>7)	1.56 (0.88-2.74)	1.65 (0.94-2.87)	1.63 (0.93-2.85)
Survey-based measure			
Workload domain			
Physical function: limitations in activities of daily living (for a 1-unit increase)			1.06 (0.99-1.13)
Chaotic lifestyle score			1.03 (1.00-1.06)
Capacity/resilience domain			
Low resilience, score			
>3			1 [Reference]
≤3			0.81 (0.65-1.02)
Married			
No			1 [Reference]
Yes			0.75 (0.62-0.92)
Preference/expectations			
Powerful others health-related locus of control (score)			
≤18			1 [Reference]
>18 to 22			1.05 (0.84-1.30)
>22 to 30			0.78 (0.59-1.03)
Mean AIC (SD)	2981.9 (2.8)	2961.31(2.5)	2951.9 (2.2)

Abbreviations: AIC, Akaike Information Criterion; AUDIT-C, Alcohol Use Disorders Identification Test; BMI, body mass index (calculated as weight in kilograms divided by height in meters squared); EHR, electronic health record; VA, Veterans Affairs.

^a^ Calculated using PROC MIANALYZE within SAS software, version 9.4, to combine estimates from 10 imputed data sets (SAS PROC MI) per model type. Although the primary analyses were not based on statistical significance, the 95% CIs are provided to inform uncertainty around the magnitude of the estimates from the covariates selected as improving estimates of the outcomes based on AIC.

^b^ Gagne score was included in the model as a piecewise continuous variable comprised of 2 pieces, one for Gagne scores with initial value of −2 to 0 and the other for Gagne scores with initial values of 1 to 13. The reported Gagne odds ratios and 95% CIs are for a 1-unit increase in the Gagne score.

## Discussion

In this study of a nationally representative sample of high-risk veterans, augmenting EHR data with patient-reported SDH measures consistently improved estimates of 90-day and 180-day hospital admission risk, based on substantial reduction in AIC, compared with models with EHR-based covariates. Our findings contribute to a growing body of literature examining the association between SDH factors and hospitalization, including studies highlighting the critical roles of social support and neighborhood socioeconomic status (SES).^3,50,51,52^

This study has implications for the National Academy of Medicine (previously the Institute of Medicine) recommendations^53,54^ for the routine integration of certain SDH factors into the EHR.^9,10,55^ One of the measures that improved estimates of hospitalization in our models (smoking status) is in the list recommended by the National Academy of Medicine.^55^ Our findings suggest that adding factors such as physical function, marital or partner status, and medication insecurity to annual preventive health screenings could offer an opportunity to improve prediction algorithms. Routine collection of these risk factors would enable the VA to flag patients at heightened risk for hospitalization due to these factors and enable EHR-based alerts for clinicians about individuals who need supportive services. Incorporating these types of factors into the EHR could also assist with population management and health system decisions, for example by highlighting the need for partnerships with certain community agencies.^8,56,57^

Additional implementation considerations are important when applying these findings. First, different screening instruments may be needed to address key SDH factors and disparities in specific populations, and validation of SDH measures in diverse populations is necessary. There are a growing number of brief screening instruments available, such as PRAPARE,^58,59^ THRIVE,^8^ and the Centers for Medicare & Medicaid Services Accountable Health Communities screening tool,^60^ several of which are being tested in clinics serving vulnerable patient populations. Second, although there are potential benefits to more standardized assessment of SDH factors in clinical settings, there is also a need to explore some of the potentially negative nuances.^61^ Screening for SDH factors carries potential for inappropriate treatment choices based on inadequate decision support around SDH information or risk of discrimination.^62,63^ In addition, clinicians in many health care settings may not have the comfort or capacity to address SDHs^64^; thus, integration of these efforts with community and government resources is critical.^65^ Finally, there are significant (cumulative) time and financial costs for collecting SDH measures, so only those associated with important clinical or economic outcomes should be adopted.

### Limitations

There are several limitations to this analysis that must be acknowledged. First, this study focused on individuals at high risk for hospitalization; thus, the results may not generalize to the entire VA and, therefore, cannot address how to improve estimation of hospitalization risk for the entire VA population. Second, respondents differed from nonrespondents in terms of hospitalization rate (10.5% vs 13.5%) and mortality rate (1.9% vs 5.3%) at 180 days. Veterans needed to have a valid mailing address to participate in the study. Thus, individuals who are homeless or have unstable housing are underrepresented. Women, minorities, and veterans who are younger or reside in rural locations also appeared to be underrepresented, but these were the only 3 of 36 covariates that were severely imbalanced, suggesting that the selection bias on observables was modest overall. In addition, owing to data availability at the time of analyses, hospitalizations were limited to those that took place in the VA system and do not capture hospitalizations that were covered by Medicare, Medicaid, or private insurance. Future work on more common outcomes is needed to understand whether these same covariates (or others) are associated with outcomes that matter most to veterans and the VA health system.

## Conclusions

In conclusion, we found that integration of certain patient-reported SDH measures improved estimates of 90-day and 180-day hospitalization risk among veterans with a high risk for hospitalization. Our findings contribute to ongoing discussions about SDH measures that might be of greatest value for screening efforts in the clinical setting. Incorporating select measures into clinical care and the EHR could help identify individuals at high risk for hospitalization who might benefit from targeted support.

## Notes

Supplement. **eFigure 1.** Number of Times (Out of 10 Imputations) That Each Covariate Was Chosen Through Forward Selection for Inclusion in Final Primary Analysis Logistic Regression Models **eFigure 2.** Number of Times (Out of 10 Imputations) That Each Covariate Was Chosen Through Forward Selection for Inclusion in Final Post Hoc Logistic Regression Models **eFigure 3.** Flow Diagram of Analytic Cohort **eTable 1.** Odds Ratios and 95% Confidence Intervals for Post Hoc Logistic Regression Associations Between Covariates of Interest and 90-Day Hospital Admission, by Model Type (n=4,685) **eTable 2.** Odds Ratios and 95% Confidence Intervals for Post Hoc Logistic Regression Associations Between Covariates of Interest and 180-Day Hospital Admission, by Model Type (n=4,685)
