# Supplementary material for: Patient-Reported Social and Behavioral Determinants of Health and Estimated Risk of Hospitalization in High-Risk Veterans Affairs Patients
Source: JAMA Netw Open. 2020 Oct 20;3(10):e2021457. doi: 10.1001/jamanetworkopen.2020.21457 (PMC7576406; doi:10.1001/jamanetworkopen.2020.21457)
Supplement: Supplement. — eFigure 1. Number of Times (Out of 10 Imputations) That Each Covariate Was Chosen Through Forward Selection for Inclusion in Final Primary Analysis Logistic Regression Models eFigure 2. Number of Times (Out of 10 Imputations) That Each Covariate Was Chosen Through Forward Selection for Inclusion in Final Post Hoc Logistic Regression Models eFigure 3. Flow Diagram of Analytic Cohort eTable 1. Odds Ratios and 95% Confidence Intervals for Post Hoc Logistic Regression Associations Between Covariates of Interest and 90-Day Hospital Admission, by Model Type (n=4,685) eTable 2. Odds Ratios and 95% Confidence Intervals for Post Hoc Logistic Regression Associations Between Covariates of Interest and 180-Day Hospital Admission, by Model Type (n=4,685) [file jamanetwopen-e2021457-s001.pdf]

Supplementary Online Content

Zulman DM, Maciejewski ML, Grubber JM, et al. Patient-reported social and behavioral determinants of health and estimated risk of hospitalization in high-risk Veterans Affairs patients. *JAMA Netw Open*. 2020;3(10):e2021457. doi:10.1001/jamanetworkopen.2020.21457

eFigure 1. Number of Times (Out of 10 Imputations) That Each Covariate Was Chosen Through Forward Selection for Inclusion in Final Primary Analysis Logistic Regression Models

eFigure 2. Number of Times (Out of 10 Imputations) That Each Covariate Was Chosen Through Forward Selection for Inclusion in Final Post Hoc Logistic Regression Models

eFigure 3. Flow Diagram of Analytic Cohort

eTable 1. Odds Ratios and 95% Confidence Intervals for Post Hoc Logistic Regression Associations Between Covariates of Interest and 90-Day Hospital Admission, by Model Type (n=4,685)

eTable 2. Odds Ratios and 95% Confidence Intervals for Post Hoc Logistic Regression Associations Between Covariates of Interest and 180-Day Hospital Admission, by Model Type (n=4,685)

This supplementary material has been provided by the authors to give readers additional information about their work.

© 2020 Zulman DM et al. *JAMA Network Open*.

eFigure 1. Number of Times (Out of 10 Imputations) That Each Covariate Was Chosen Through Forward Selection for Inclusion in Final Primary Analysis Logistic Regression Models

Outcome = 90-day All-Cause Admission to VA Hospital

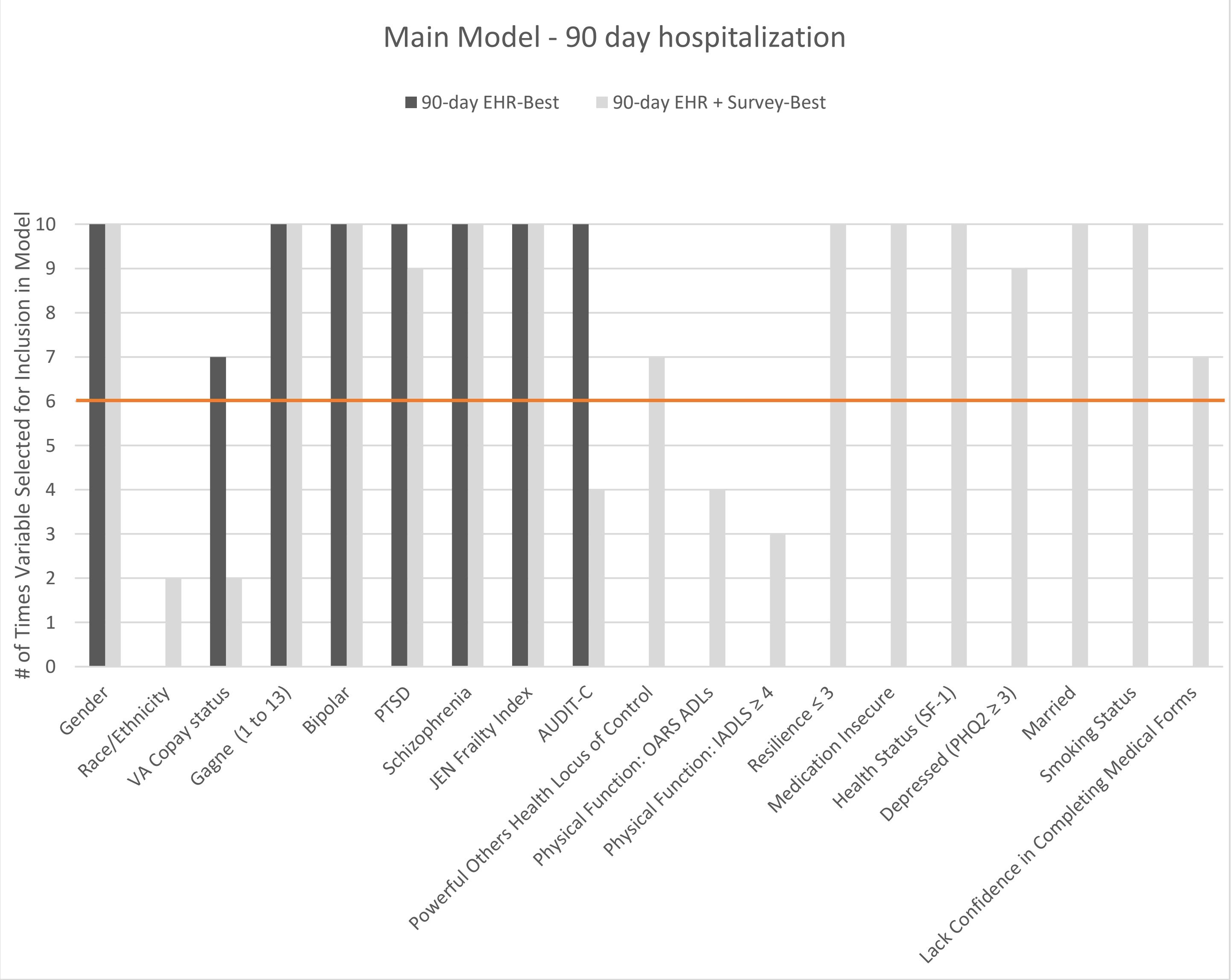

© 2020 Zulman DM et al. *JAMA Network Open*.

Outcome = 180-day All-Cause Admission to VA Hospital

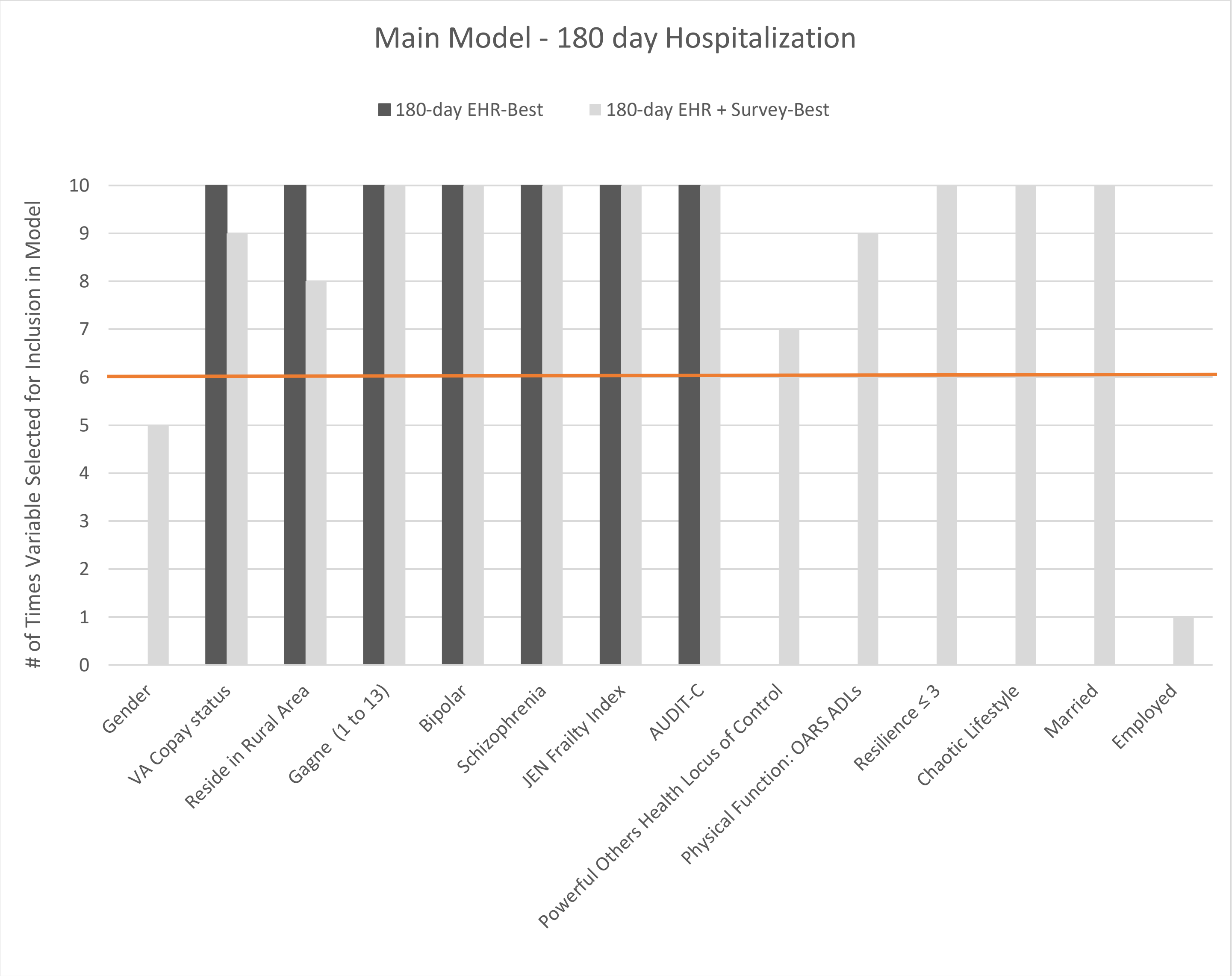

© 2020 Zulman DM et al. *JAMA Network Open*.

eFigure 2. Number of Times (Out of 10 Imputations) That Each Covariate Was Chosen Through Forward Selection for Inclusion in Final Post Hoc Logistic Regression Models

Outcome = 90 Day All-Cause Admission to VA Hospital

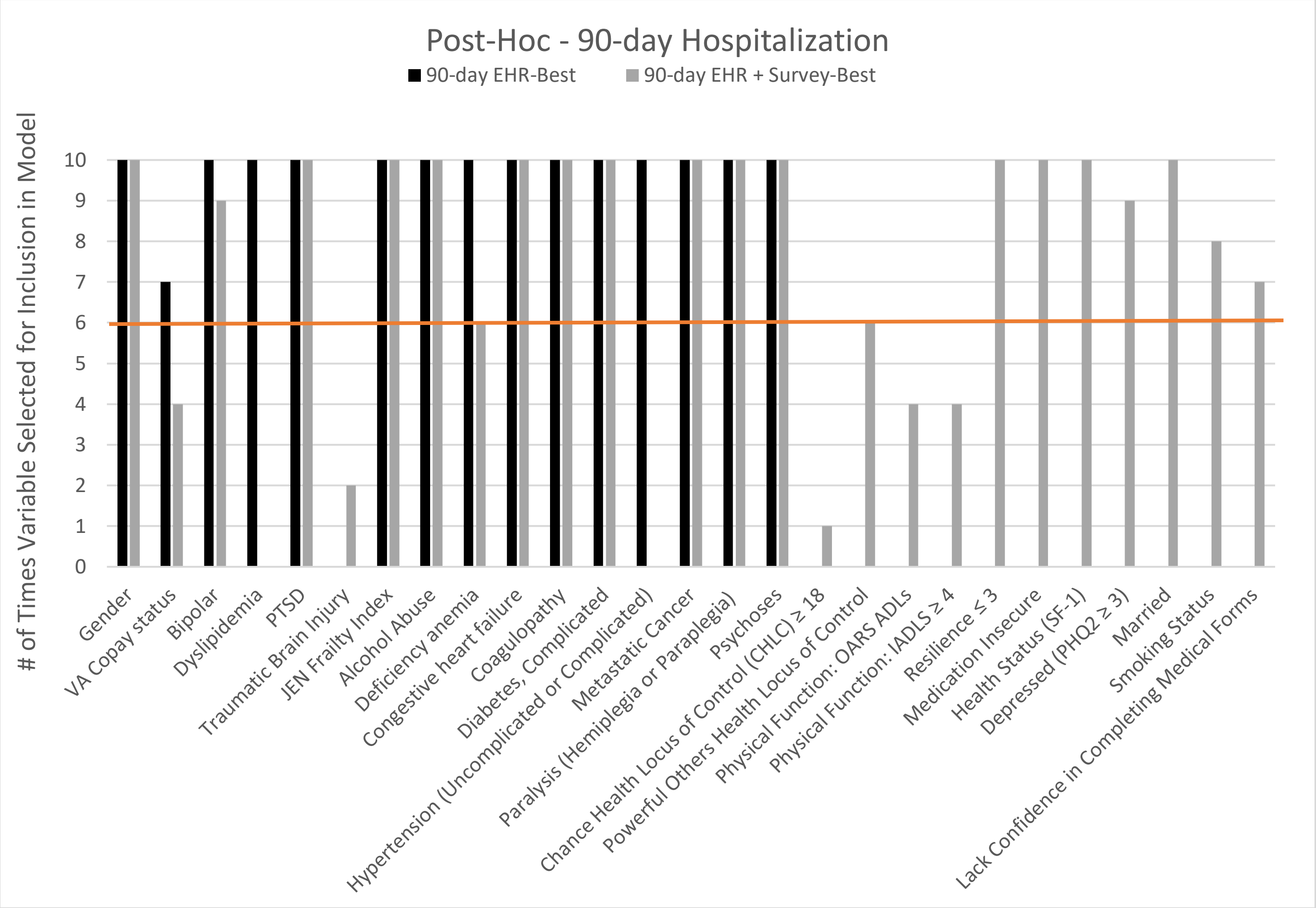

© 2020 Zulman DM et al. *JAMA Network Open*.

Outcome = 180-day All-Cause Admission to VA Hospital

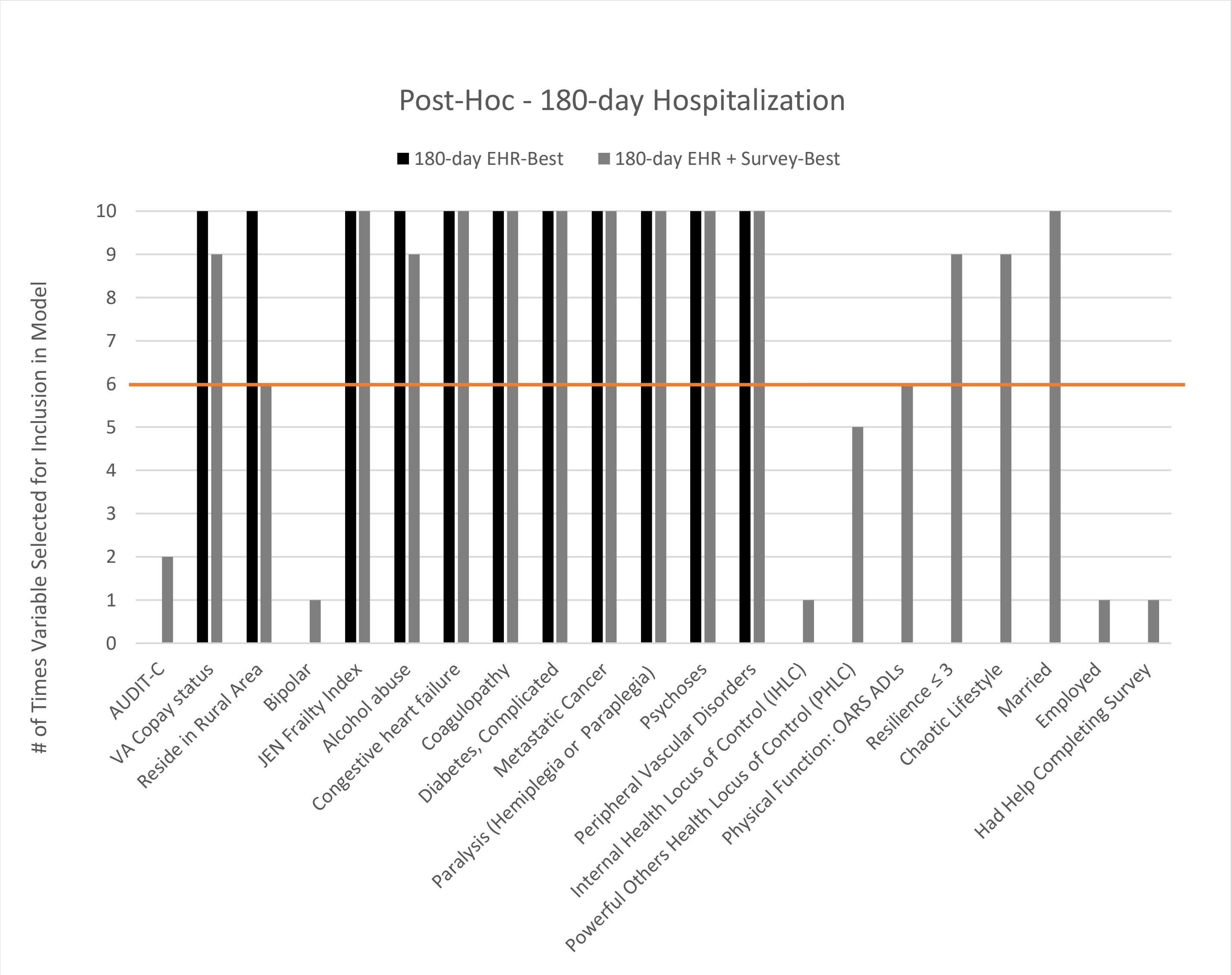

© 2020 Zulman DM et al. *JAMA Network Open*.

eFigure 3. Flow Diagram of Analytic Cohort

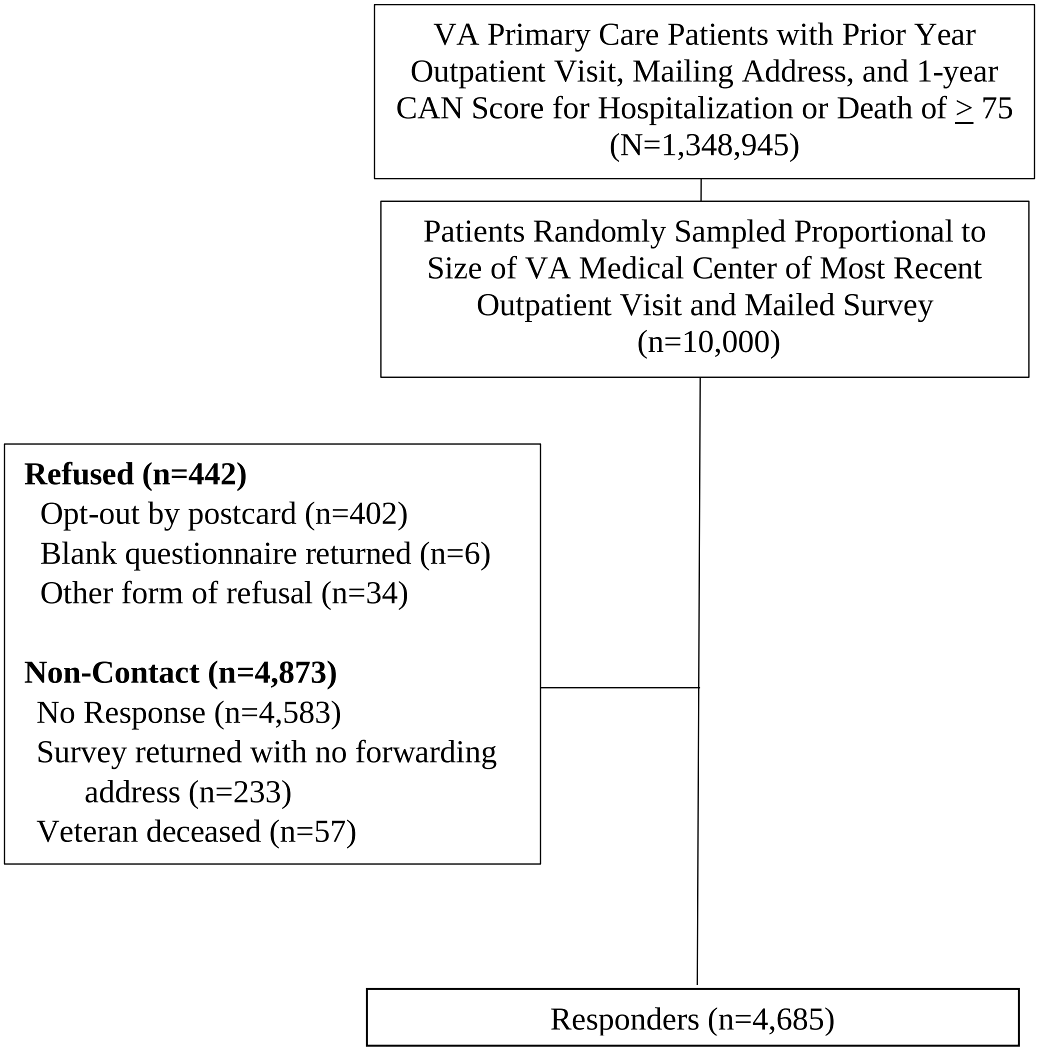

© 2020 Zulman DM et al. *JAMA Network Open*.

eTable 1. Odds Ratios and 95% Confidence Intervals for Post Hoc Logistic Regression Associations Between Covariates of Interest and 90-Day Hospital Admission, by Model Type (n=4,685)

Covariates	*Model with EHR-based covariates*		*Model with EHR- and survey-based covariates*
	All Odds Ratio[a] (95% CI)	Forward-selected Odds Ratio[a] (95% CI)	Forward-selected Odds Ratio[a] (95% CI)
**EHR-Based Measures**			
**Demographics**			
Age			
<60	1.00 (reference)		
60-80	1.04 (0.68-1.59)		
>80	0.98 (0.57-1.68)		
Gender (Female vs Male (ref))	0.57 (0.27-1.19)	0.54 (0.26-1.12)	0.51 (0.25-1.07)
Race/Ethnicity			
White non-Hispanic	1.00 (reference)		
Black non-Hispanic	1.05 (0.74-1.50)		
Hispanic	1.57 (0.91-2.72)		
Other	1.14 (0.50-2.57)		
VA Copay Status (Non-exempt vs. Exempt (ref))	0.75 (0.49-1.13)	0.74 (0.49-1.10)	
Rural Residence (vs. Non-rural (ref))	0.88 (0.66-1.16)		
**Chronic Health Conditions**			
Gagne Comorbidity Score Individual Components (Presence vs. Absence (ref))			
Alcohol abuse	1.39 (0.90-2.13)	1.67 (1.14-2.42)	1.49 (1.01-2.20)
Deficiency anemia	0.62 (0.35-1.10)	0.65 (0.37-1.15)	0.67 (0.38-1.18)
Arrhythmia	0.96 (0.69-1.33)		
Congestive heart failure	1.28 (0.89-1.84)	1.31 (0.94-1.82)	1.34 (0.96-1.87)
Coagulopathy	1.75 (0.88-3.51)	1.79 (0.90-3.53)	1.78 (0.90-3.55)
Chronic obstructive pulmonary disease	1.10 (0.83-1.45)		
Dementia	0.92 (0.49-1.70)		
Diabetes, complicated	1.25 (0.93-1.67)	1.23 (0.93-1.62)	1.27 (0.97-1.67)
Fluid and electrolyte disorders	1.05 (0.67-1.64)		
HIV/AIDS	1.12 (0.25-5.00)		
Hypertension (Uncomplicated or complicated)	1.30 (0.92-1.82)	1.30 (0.93-1.80)	
Liver Disease	1.11 (0.70-1.75)		
Metastatic Cancer	1.98 (0.93-4.18)	1.95 (0.96-3.96)	1.94 (0.95-3.96)
Paralysis (hemiplegia or paraplegia)	7.01 (1.64-29.92)	3.46 (1.48-8.13)	3.35 (1.42-7.94)
Psychoses	1.90 (0.55-6.62)	2.17 (1.20-3.91)	2.06 (1.13-3.74)
Pulmonary/Circulation	0.90 (0.39-2.05)		

© 2020 Zulman DM et al. *JAMA Network Open*.

Peripheral vascular disorders	1.27 (0.90-1.78)		
Renal Failure	0.86 (0.60-1.22)		
Any Malignancy	1.01 (0.72-1.42)		
Weight Loss	1.14 (0.61-2.15)		
Non-Gagne Health Conditions (Presence vs. Absence (ref))			
Anxiety	0.92 (0.63-1.36)		
Bipolar Disorder	1.72 (0.94-3.16)	1.63 (0.90-2.93)	1.56 (0.86-2.84)
Dyslipidemia	0.79 (0.61-1.04)	0.79 (0.60-1.03)	
Post Traumatic Stress Disorder	0.68 (0.47-0.97)	0.67 (0.47-0.95)	0.72 (0.50-1.03)
Schizophrenia	1.11 (0.28-4.45)		
Traumatic Brain Injury	2.20 (0.45-10.74)		
Spinal Cord Trauma	0.37 (0.06-2.24)		
**Other Clinical Characteristics**			
BMI			
<25	1.00 (reference)		
25 - <30	0.96 (0.65-1.42)		
30 - <35	0.91 (0.60-1.39)		
≥35	1.00 (0.65-1.55)		
Frailty (JEN Frailty Index)	1.25 (1.16-1.36)	1.25 (1.16-1.34)	1.24 (1.16-1.33)
Alcohol Use (AUDIT-C Score)			
None (0)	1.00 (reference)		
Low (1-2 (female), 1-3 (male))	0.96 (0.71-1.30)		
Moderate (3-7 (female), 4-7 (male))	1.13 (0.69-1.84)		
High (>7)	1.88 (0.93-3.81)		
**Survey-Based Measures**			
**Workload Domain**			
Global Health Status (Good/Very Good/Excellent vs. Poor/Fair (ref))			1.35 (1.01-1.80)
Tobacco Use			
Never smoker			1.00 (reference)
Past smoker			1.13 (0.81-1.57)
Current, less than daily, smoker			0.87 (0.44-1.73)
Current daily smoker			1.57 (1.06-2.34)
Medication Insecure (vs. Secure (ref))			0.63 (0.40-1.00)
Depression Symptoms (PHQ2 ≥3 vs. <3 (ref))			1.30 (0.95-1.78)
**Capacity/Resilience Domain**			
Lack Confidence in Completing Medical Forms)			1.34 (0.92-1.94)
Resilience (score ≤3 vs. > 3 (ref))			0.73 (0.54-0.98)
Married (vs. Not Married (ref))			0.69 (0.52-0.90)
**Preference/Expectations[b]**			
Powerful Others Health Locus of Control (score)			

© 2020 Zulman DM et al. *JAMA Network Open*.

≤ 18			1.00 (reference)
>18-22			1.04 (0.78-1.38)
>22			0.73 (0.49-1.06)
**Mean AIC (SD)**	1994.13 (1.49)	1953.99 (1.32)	1940.24 (2.48)

[a] Odd ratios for each model type were calculated using PROC MIANALYZE within SAS software (v9.4) to combine estimates from 10 imputed data sets (SAS PROC MI) per model type. While the analyses were not based on statistical significance, the 95% CIs are provided to inform uncertainty around the magnitude of the estimates from the covariates selected as improving prediction of the outcomes based on AIC.

© 2020 Zulman DM et al. *JAMA Network Open*.

eTable 2. Odds Ratios and 95% Confidence Intervals for Post Hoc Logistic Regression Associations Between Covariates of Interest and 180-Day Hospital Admission, by Model Type (n=4,685)

	Model with EHR-based covariates		*Model with EHR- and survey-based covariates*
	All Odds Ratio[a] (95% CI)	Forward-selected Odds Ratio[a] (95% CI)	Forward-selected Odds Ratio[a] (95% CI)
**EHR-Based Measures**			
**Demographics**			
Age			
<60	1.00 (reference)		
60-80	0.87 (0.64-1.19)		
>80	0.78 (0.52-1.17)		
Gender (Female vs. Male (ref))	0.79 (0.49-1.27)		
Race/Ethnicity	1.00 (reference)		
White non-Hispanic	0.95 (0.72-1.26)		
Black non-Hispanic	1.04 (0.64-1.67)		
Hispanic	1.27 (0.70-2.30)		
VA Copay Status (Non-exempt vs. Exempt (ref))	0.69 (0.50-0.97)	0.70 (0.51-0.97)	0.74 (0.53-1.02)
Rural Residence (vs. Non-rural (ref))	0.84 (0.68-1.04)	0.85 (0.69-1.05)	0.86 (0.70-1.06)
**Chronic Health Conditions**			
Gagne Comorbidity Score Individual Components (Presence vs. Absence (ref))			
Alcohol abuse	1.23 (0.87,1.72)	1.46 (1.08-1.96)	1.42 (1.05-1.92)
Deficiency anemia	0.96 (0.65-1.40)		
Arrhythmia	1.07 (0.83-1.36)		
Congestive heart failure	1.36 (1.03-1.79)	1.41 (1.09-1.81)	1.41 (1.09-1.83)
Coagulopathy	2.25 (1.35-3.76)	2.23 (1.34-3.70)	2.34 (1.41-3.89)
Chronic obstructive pulmonary disease	0.98 (0.79-1.22)		
Dementia	1.00 (0.63-1.60)		
Diabetes, complicated	1.23 (0.98-1.54)	1.22 (1.00-1.51)	1.24 (1.01-1.53)
Fluid and electrolyte disorders	1.10 (0.78-1.56)		
HIV/AIDS	0.63 (0.14-2.71)		
Hypertension (Uncomplicated or complicated)	1.09 (0.85-1.40)		
Liver Disease	1.09 (0.76-1.57)		
Metastatic Cancer	1.66 (0.87-3.15)	1.64 (0.89-3.03)	1.68 (0.91-3.10)
Paralysis (hemiplegia or paraplegia)	3.98 (0.99-16.03)	2.54 (1.18-5.44)	2.48 (1.14-5.40)
Psychoses	1.51 (0.56-4.10)	1.92 (1.19-3.09)	1.83 (1.13-2.97)
Pulmonary/Circulation	1.25 (0.69-2.26)		
Peripheral vascular disorders	1.26 (0.97-1.65)	1.29 (1.00 ,1.67)	1.28 (0.99-1.66)
Renal Failure	0.97 (0.74 -1.27)		
Any Malignancy	0.89 (0.68 -1.16)		
Weight Loss	1.27 (0.78 -2.06)		

© 2020 Zulman DM et al. *JAMA Network Open*.

Non-Gagne Health Conditions (Presence vs. Absence (ref))			--
Anxiety	0.94 (0.70-1.26)		
Bipolar Disorder	1.41 (0.87-2.29)		
Dyslipidemia	0.93 (0.75-1.15)		
Post Traumatic Stress Disorder	0.87 (0.67-1.13)		
Schizophrenia	1.23 (0.40-3.78)		
Traumatic Brain Injury	1.09 (0.22-5.33)		
Spinal Cord Trauma	0.50 (0.10-2.61)		
**Other Clinical Characteristics**			
BMI			
<25	1.00 (reference)		
25 - <30	0.92 (0.69-1.24)		
30 - <35	0.90 (0.66-1.24)		
≥35	0.96 (0.69-1.34)		
Frailty (JEN Frailty Index, for 1-unit increase)	1.22 (1.15-1.30)	1.22 (1.16-1.29)	1.22 (1.15-1.29)
Alcohol Use (AUDIT-C Score)			
None (0)	1.00 (reference)		
Low (1-2 (female), 1-3 (male))	0.90 (0.71-1.14)		
Moderate (3-7 (female), 4-7 (male))	1.27 (0.89-1.83)		
High (> 8)	1.45 (0.79-2.66)		
**Survey Measures**			
**Workload Domain**			
Physical Function: Limitations in Activities of Daily Living			1.04 (0.98-1.12)
Chaotic Lifestyle Score (for 1-unit increase)			1.03 (1.00-1.06)
**Capacity/Resilience Domain**			
Resilience (score ≤3 vs. > 3 (ref))			0.81 (0.65-1.01)
Married (vs. Not Married (ref))			0.75 (0.61-0.92)
**Preference/Expectations**[b]			
**Mean AIC (SD)**	2994.78 (2.52)	2949.87 (2.26)	2941.35 (2.14)

[a] Odd ratios for each model type were calculated using PROC MIANALYZE within SAS software (v9.4) to combine estimates from 10 imputed data sets (SAS PROC MI) per model type. While the analyses were not based on statistical significance, the 95% CIs are provided to inform uncertainty around the magnitude of the estimates from the covariates selected as improving prediction of the outcomes based on AIC.

[b] Covariates from this domain were not selected in at least 6 of the 10 imputed data set logistic regression models; therefore none were included in the final 180-day post-hoc EHR- and survey-based models.

© 2020 Zulman DM et al. *JAMA Network Open*.
